# NovoGraph: Human genome graph construction from multiple long-read *de novo* assemblies

**DOI:** 10.12688/f1000research.15895.2

**Published:** 2018-12-10

**Authors:** Evan Biederstedt, Jeffrey C. Oliver, Nancy F. Hansen, Aarti Jajoo, Nathan Dunn, Andrew Olson, Ben Busby, Alexander T. Dilthey

**Affiliations:** 1 Weill Cornell Medicine, New York, NY, 10065, USA; 2 New York Genome Center, New York, NY, 10013, USA; 3 Office of Digital Innovation and Stewardship, University Libraries, University of Arizona, Tucson, AZ, 85721, USA; 4 National Human Genome Research Institute, National Institutes of Health, Bethesda, MD, 20817, USA; 5 Baylor College of Medicine, Houston, TX, 77030, USA; 6 Lawrence Berkeley National Laboratory, Berkeley, CA, 94720, USA; 7 Cold Spring Harbor Laboratory, Cold Spring Harbor, NY, 11724, USA; 8 National Center for Biotechnology Information, National Institutes of Health, Bethesda, MD, 20817, USA; 9 Institute of Medical Microbiology and Hospital Hygiene, Heinrich Heine University Düsseldorf, Düsseldorf, 40225, Germany

**Keywords:** Genome graph, de novo assembly, alignment, multiple sequence alignment, population reference graph, NovoGraph

## Abstract

Genome graphs are emerging as an important novel approach to the analysis of high-throughput human sequencing data. By explicitly representing genetic variants and alternative haplotypes in a mappable data structure, they can enable the improved analysis of structurally variable and hyperpolymorphic regions of the genome. In most existing approaches, graphs are constructed from variant call sets derived from short-read sequencing. As long-read sequencing becomes more cost-effective and enables *de novo *assembly for increasing numbers of whole genomes, a method for the direct construction of a genome graph from sets of assembled human genomes would be desirable. Such assembly-based genome graphs would encompass the wide spectrum of genetic variation accessible to long-read-based *de novo* assembly, including large structural variants and divergent haplotypes.

Here we present NovoGraph, a method for the construction of a human genome graph directly from a set of *de novo* assemblies. NovoGraph constructs a genome-wide multiple sequence alignment of all input contigs and creates a graph by merging the input sequences at positions that are both homologous and sequence-identical. NovoGraph outputs resulting graphs in VCF format that can be loaded into third-party genome graph toolkits. To demonstrate NovoGraph, we construct a genome graph with 23,478,835 variant sites and 30,582,795 variant alleles from *de novo* assemblies of seven ethnically diverse human genomes (AK1, CHM1, CHM13, HG003, HG004, HX1, NA19240). Initial evaluations show that mapping against the constructed graph reduces the average mismatch rate of reads from sample NA12878 by approximately 0.2%, albeit at a slightly increased rate of reads that remain unmapped.

## Introduction

Since the release of the first complete version of the human reference genome in 2003, large-scale genomic sequencing has been established as a key tool for both fundamental research and personalized medicine. Sequencing costs have fallen dramatically, and the whole genomes of tens of thousands of individuals have been sequenced and analyzed. Although long-read sequencing is becoming more cost-effective and popular, the sequencing technologies that currently dominate cohort sequencing produce millions of short reads between 100 and 250 base pairs in length. As the first step of data analysis, these reads are typically mapped to the human reference genome to determine their genomic locations.

This approach works well for the large majority of reads; critically, however, it fails for reads that come from regions in the sequenced genome that are strongly divergent from the reference genome. Important examples include immunogenetic regions known to harbor important disease-associated variants like the major histocompatibility complex (MHC) and the killer-cell immunoglobulin-like receptor (KIR) genes ( Kuśnierczyk, 2013; Trowsdale & Knight, 2013), as well as regions affected by large or complex structural variants, which together account for more than 50% of total base pair differences between individuals ( Sudmant *et al.*, 2015). The total proportion of the human genome inaccessible to classical reference-based analysis is estimated to be greater than 1% ( Dilthey *et al.*, 2015).

Instead of mapping reads to a single reference genome, it is now possible to map reads to a reference genome graph ( Computational Pan-Genomics Consortium, 2018; Paten *et al.*, 2017; Schneeberger *et al*., 2009). A reference genome graph can be thought of as a data structure that provides a unified representation of multiple genomes from the same species. As more genomes are added to the graph, the probability that any given region in a sequenced genome has a sufficiently close homolog in the graph (so as to allow for reliable mapping) increases. Technically, a genome graph is an acyclic or cyclic graph structure with nucleotide-labeled edges or nodes; each input genome can typically be reconstructed as a traversal of the graph, and nodes with more than one incoming or outgoing edge represent transition points between the input genomes. Like linear reference genomes, genome graphs can serve as the basis for read mapping and variant calling.

The utility of reference genome graphs in the field of human genetics was first demonstrated in the field of immunogenetics and subsequently for the entire human genome. Specifically, a reference graph approach to model local haplotype structures enabled improved genotyping accuracy in the MHC ( Dilthey *et al.*, 2015) and, for the first time, reliable typing of the Human Leukocyte Antigen (HLA) genes from standard whole-genome sequencing data ( Dilthey *et al.*, 2016). More recently, multiple graph approaches and software toolkits suitable for genome-wide application have been published ( Eggertsson *et al.*, 2017; Garrison *et al*., 2018; Rakocevic *et al.*, 2017; Sibbesen *et al.*, 2018), showing, for example, that graph genome approaches can enable a fivefold reduction of missed SNP calls ( Eggertsson *et al.*, 2017) and enable the genotyping of thousands of additional variants longer than 50 base pairs per genome ( Sibbesen *et al.*, 2018).

These developments notwithstanding, the field is still in its infancy. One particularly important open question is how to integrate information from long-read sequencing into the graph construction process. In existing approaches, graph construction typically relies on call sets derived from short-read sequencing experiments. As discussed above, however, short-read sequencing has limited sensitivity in the hypervariable and structural-variation-rich regions where graph genomes can be expected to provide the greatest benefit. Therefore, graphs constructed via existing methods likely miss substantial proportions of relevant variation. By contrast, long-read-sequencing enables the assembly of complex sequences ( Jain *et al.*, 2018) in a reference-bias-free way and the detection of structural variants at high sensitivity ( Sedlazeck *et al.*, 2018). Even though the number of long-read-sequenced samples is still limited, rendering their sequences available via a genome graph would be highly desirable.

Here we introduce NovoGraph, a pipeline for the direct construction of acyclic genome graphs from *de novo* assembly contigs. NovoGraph constructs a whole-genome graph by merging the input assembly sequences at positions of homology. This approach has the advantage that the resulting graph will generally include the complete set of sequences present in the input assemblies, including (at base-pair resolution) the sequences that correspond to structural variants and divergent haplotypes. Graphs constructed by NovoGraph will therefore be comparatively enriched in large-scale structural and complex variants. In the spirit of modularity, constructed graphs are represented in VCF format, which enables them to be used with any of the established genome-wide graph toolkits. We also utilize the standard CRAM format for representing the output of intermediate steps, in particular a multiple sequence alignment of all input sequences.

The genome graph construction problem is related to other algorithms and approaches to establish homology relationships between sets of sequences, for example multiple sequence alignment ( Bradley *et al*., 2009; Edgar, 2004; Katoh & Standley, 2013; Lassmann & Sonnhammer, 2005; Notredame *et al*., 2000; Sievers & Higgins, 2014; Thompson *et al*., 1994), whole-genome alignment ( Angiuoli & Salzberg, 2011; Blanchette *et al*., 2004; Darling *et al*., 2004; Darling *et al*., 2010; Höhl *et al*., 2002; Salazar & Abeel, 2018), A-Bruijn alignment ( Raphael *et al*., 2004), or Cactus ( Paten *et al*., 2011). Multiple sequence alignment algorithms are not directly applicable to the problem of constructing whole-genome graphs from multiple *de novo* assemblies; they don’t directly support multi-chromosomal alignment scenarios and typically don’t scale to aligning thousands of contigs across multi-gigabase genomes. Methods and data structures for whole-genome alignment, on the other hand, are typically designed to detect homology relationships between more distantly related genomes of different species and explicitly model complex large-scale events, such as chromosomal inversions and translocations, in terms of a (potentially cyclic) sequence graph. Although these data structures describe essential aspects of genome evolution, such whole-genome alignment graphs are not supported by the alignment and/or inference modules of the existing genome-wide graph toolkits. By contrast, NovoGraph is designed to construct graphs that are universally compatible with existing downstream software for graph-based genome inference, and uses a localization approach to enable the application of existing multiple sequence alignment algorithms in the context of graph construction (described in detail below).

We demonstrate NovoGraph by constructing a genome graph from seven ethnically diverse human genomes and the canonical reference. In a mapping experiment with vg ( Garrison *et al*., 2018), we show that using this graph instead of the standard reference genome increases the average alignment identity of genome-wide short reads.

This project was initiated at an NCBI hackathon ( Busby *et al.*, 2016) held before the 2016 Biological Data Science meeting at Cold Spring Harbor Laboratory (CSHL) in October, 2016. The seven co-authors gathered for 3 days at CSHL to quickly develop and prototype the pipeline. As with all NCBI hackathons, the only stipulations for the event were (1) that the data be publicly available and (2) that any resulting software be open-source.

## Methods

### Pipeline overview

**Figure 1.**
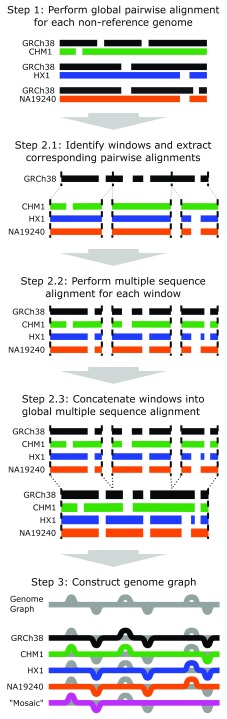
NovoGraph overview. Overview of the genome graph generation pipeline presented here. In Step 1, each genome is aligned to a reference genome (GRCh38, shown here in black). In Step 2.1, the pairwise alignments are partitioned into smaller windows for multiple sequence alignment in Step 2.2. Multiple sequence alignments are concatenated into an approximate global single alignment in Step 2.3. Finally, in Step 3, the multiple sequence alignment is converted to a single graph representation of the genome, shown in gray. Each individual genome has a single, acyclic path through the genome graph (black, green, blue, and orange paths). The magenta path represents a “mosaic” genome—that is, a path through the graph which was not observed in any genome.

The NovoGraph pipeline ( Biederstedt *et al*., 2018) for constructing a genome graph from a set of assembly contigs consists of the following steps (see Figure 1):

1. For each input contig, compute a global pairwise alignment to the GRCh38 primary assembly. This alignment determines the approximate placement of each input contig relative to the reference.
2. Compute an approximate global multiple sequence alignment (MSA) between all input contigs and the reference genome. This multiple sequence alignment embodies the joint sequence homology relationships between all input sequences and the reference genome. The pairwise contig-to-reference alignments from Step 1 are used to guide this process.
3. Compute a directed acyclic graph (DAG) from the global MSA, connecting contigs at positions that are both homologous and sequence-identical.

The outputs from Steps 1 and 2 are represented in SAM/CRAM format ( Hsi-Yang Fritz *et al.*, 2011; Li *et al.*, 2009). The output from Step 3 is a VCF ( Danecek *et al.*, 2011), which may be provided as input to various existing graph genome frameworks.

### Step 1 – Pairwise global alignments between individual input contigs and GRCh38

**Figure 2.**
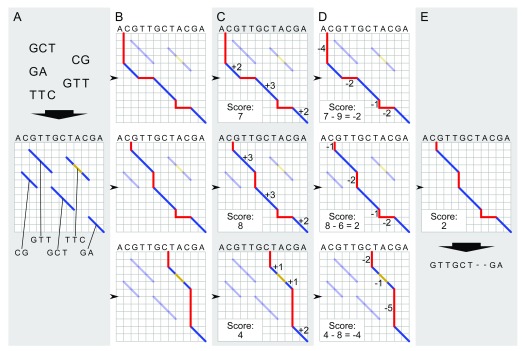
Global pairwise alignment schematic. Schematic of modified Needleman-Wunsch algorithm for global alignment of an input contig to a reference genome. The process starts with local alignments between the contig and the reference genome (blue diagonals in **A**). All possible combinations of these local alignments are enumerated by realizing all paths connecting contigs from the upper left to lower right corner of the matrix ( **B**). Each alignment is scored: matches contribute positive scores (dark blue lines in **C**), while indels (red) and mismatches (gold) incur a penalty ( **D**). The alignment with the highest score is selected as the best global alignment ( **E**) for the next step in graph genome creation; ties among global alignments are resolved arbitrarily.

For each input contig, we compute a global pairwise alignment between the input contig sequence and the GRCh38 primary assembly. This process is illustrated in Figure 2.

Exact global alignment scales quadratically with the length of the input sequences and therefore quickly becomes computationally intractable as the input sequences increase in size. We therefore adopt a heuristic approach:

First, we use bwa-mem ( Li, 2013) to identify high-scoring local alignments between the input contig and the reference genome (GRCh38). These represent diagonal (or near-diagonal) moves in a global alignment matrix, i.e. regions of high pairwise alignment identity between the input contig and a reference genome. We refer to the identified local alignments as “diagonals”.

Next, to obtain a global pairwise alignment, we identify the highest-scoring consistent combination of the identified diagonals into a global alignment by dynamic programming. Note that pairwise alignments by definition comprise two sequences in defined orientations; only diagonals that align to the same reference contig in the same orientation (strandedness) can therefore contribute to a consistent global alignment.

We now give a formal definition of the algorithm. For simplicity, we assume that all identified diagonals align to the same reference contig in the same orientation; if this is not the case, the following algorithm can be executed independently for all reference contig/orientation pairs and their corresponding diagonals, and the best global alignment between the input contig and the reference genome is the best identified alignment over all considered pairs of reference contigs and orientation.

We define a set *P_ENTRY* of “path entry” points and a set *P_EXIT* of “path exit” points. Each element *(diagonal_id, (reference_coordinate, input_contig_coordinate))* of these sets consists of a diagonal identifier and a pair of coordinates that specify positions along the reference and input sequences, similar to the coordinates in the classical Needleman-Wunsch dynamic programming matrix. For example, the coordinate pair (3, 2) refers to a state in which 3 characters of the reference and 2 characters of the input sequence have been consumed. We also define the special points *ORIGIN* as *(*NA *, (0, 0))* and *TERMINUS* as *(*NA *, (n, m))*, where *n* is the length of the reference sequence, *m* is the length of the input contig ID, and “NA” stands for an undefined diagonal identifier.

**Figure 3.**
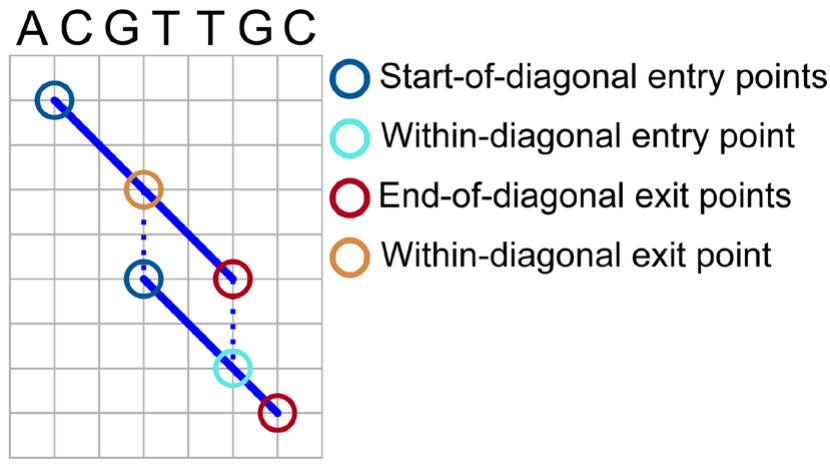
Global pairwise alignment entry points and exit points. Focus on entry and exit points for obtaining global alignments. Diagonal blue lines represent local alignments between a reference sequence and an input sequence. Circles indicate types of entry and exit points used in the algorithm to define paths through the alignment space. See text for details of algorithms and formal definitions of entry and exit points.

We populate the sets *P_ENTRY* and *P_EXIT* based on the identified diagonals. Each diagonal represents a local pairwise alignment between the reference and the input contig, and is therefore associated with two pairs of coordinates that specify the start and stop of the alignment in the reference and in the contig sequence. Specifically, let *(d _1_, d _2_)* denote the start coordinates of a given diagonal *d* in the reference and contig sequences, and let *(d _3_, d _4_)* denote the stop coordinates of the alignment in the reference and contig sequences. Both coordinate pairs are 1-based. To give an example, if diagonal *d* represents an alignment between positions 4 and 10 of the reference sequence and positions 3 and 11 of the contig sequence, *d _1_* = 4, *d _2_* = 3, *d _3_* = 10, and *d _4_* = 11. For each diagonal *d*, we add *(d, (d _1_, d _2_))* as a member of the set *P_ENTRY* and *(d, (d _3_, d _4_))* as a member of the set *P_EXIT*. We refer to these as “start-of-diagonal” entry and “end-of-diagonal” exit points. We also add “within-diagonal” path exit points that horizontally or vertically align with start-of-diagonal entry points of other diagonals, and “within-diagonal” path entry points that horizontally or vertically align with end-of-diagonal exit points of other diagonals. Specifically, we add a within-diagonal path exit point *(d, (d _x_, d _y_))* for diagonal *d* if and only if (i) the coordinates *(d _x_, d _y_)* correspond to a column in the local alignment associated with *d* and (ii) there is another diagonal *g* with *g _1 _= d _x_* or *g _2 _= d _y_*. The definition of within-diagonal path entry points follows symmetrically. The different types of entry and exit points are illustrated in Figure 3.

The set of valid path traversals is defined as the set of sequences *x _0_,x _1_,x _2_,...,x _n_* that meet the following conditions:

**(i)**  for all *i* such that *i* is even, *x _i_* is a member of { *ORIGIN* ∪ *P_EXIT*}
**(ii)**  for all *i* such that *i* is odd, *x _i_* is a member of { *TERMINUS* ∪ *P_ENTRY*}
**(iii)**  *x _0_ = ORIGIN* and *x _n _= TERMINUS*
**(iv)**  for all *x _i_=(g, (g _a_, g _b_))* and *x _i+1_= (h, (h _a_, h _b_))*, *g _a_ ≤ h _a_* and *g _b_ ≤ h _b_*
**(v)**  for all *x _i_=(g, (g _a_, g _b_))* and *x _i+1_=(h, (h _a_, h _b_))* with odd *i*, *g = h*
**(vi)**  each element of the sequence is unique.

Each traversal can be scored iteratively from left to right by combining the scores of the traversed diagonals with gap-incurred penalties from the jumps between exit and entry points. We initialize by setting *score(ORIGIN) = 0*. For odd *i* with *x _i_=(g, (g _a_, g _b_))* and *x _i-1_=(h, (h _a_, h _b_))*, we set *score(x _i_) = score(x _i-1_) + gap_score x [(h _a _- g _a_)+(h _b _- g _b_)]*. For even *i* with *x _i_=(g, (g _a_, g _b_))* and *x _i-1_=(h, (h _a_, h _b_))*, *g* is equal to *h* by definition and we set *score(x _i_)* to be *score(x _i-1_)* plus the score of the local alignment on diagonal *g* between coordinates *(h _a_,h _b_)* and *(g _a_,g _b_)*. In the current implementation, *gap_score* is -1, matches within local alignments are scored as +1, and mismatches/gaps within local alignments as -1. Jumps to *ORIGIN* and *TERMINUS* are not penalized along the reference dimension (i.e., ends-free alignment).

A dynamic programming formulation for finding the highest-scoring traversal follows immediately from these definitions. In brief, order the union set *S := {ORIGIN ∪ P_ENTRY ∪ P_EXIT ∪ TERMINUS}* by coordinates and for the *i*-th element of the ordered set *S*, compute the maximum achievable score *max_score(x _i_)* of *x _i_* by

**(i)**  identifying the subset *S’ ⊆ {x _0_ , .., x _i-1_}* of possible predecessor elements
**(ii)**  for each *s ϵ S’*, scoring the transition from *s* to *x _i_* by replacing *‘score’* with *‘max_score’* in the definitions of the preceding paragraph
**(iii)**  selecting the maximum-scoring transition as the value for *max_score(x _i_)*.

If bwa-mem fails to identify any local alignments between an input contig and the reference, it is impossible to compute an approximate global pairwise alignment, and the contig is ignored during all subsequent steps.

### Step 2 – Approximate global multiple sequence alignment

We now turn the pairwise input-contig-to-reference alignments created in Step 1 into a set of approximate global multiple sequence alignments.

We split the GRCh38 reference contigs into non-overlapping windows of approximately 10,000 bases. A window size of approximately 10,000 is chosen to be both sufficiently large to include the majority of human structural variants and small enough to allow for efficient processing of individual windows; see below for a precise definition of how window boundaries are determined. For each window, we extract the reference sequence and, based on the pairwise input-contig-to-reference alignments, the input contig sequences overlapping the window. We use MAFFT ( Katoh & Standley, 2013), selected after initial experiments for speed and stability, to generate an MSA for the sequences of each window (including the reference); other state-of-the-art tools for multiple sequence alignment, e.g. MUSCLE ( Edgar, 2004) or FSA ( Bradley *et al*., 2009), could also be employed. The per-window MSA generation step is trivially parallelizable. After having computed an MSA for each window, we concatenate the per-window MSAs in the correct order. For each GRCh38 reference contig, this yields a combined approximate MSA of the reference sequence and all input contigs initially aligned to it.

In this approach, the initial pairwise alignments determine in which window a given part of an input sequence ends up for the MSA computation. Ideally we would like to choose the window boundary positions so as to avoid regions of high uncertainty in the initial pairwise alignments. The placement of gaps in sequence alignments is often ambiguous and gaps are generally associated with increased alignment uncertainty.

We therefore adopt a simple heuristic to avoid the crossing of gaps when choosing window boundaries: First we partition the reference into windows of exactly 10,000 bases in length. For each window boundary position independently, we scan the surrounding ± 100 reference positions. For each considered reference position, we identify the columns corresponding to that reference position in the pairwise sequence alignments, and count the number of gaps across the identified columns. We then choose the considered reference position with the lowest proportion of gaps as the final window boundary. Final window sizes therefore vary between 9,800 and 10,200 bases.

The output from this step is encoded in CRAM format. Reference gaps are represented using the ‘P’ CIGAR character.

### Step 3 – Graph construction

As a last step, the multiple sequence alignment generated during the previous step is transformed into a graph. An important design decision for this operation is where to allow for merging between the input sequences, i.e. where to allow for transitions between sequences encoded on different input contigs. The applied merging rules shape the topology of the graph and determine the set of genomes that could be sampled from the graph; if the graph is interpreted as a generative model of genomic sequences, merge points can be interpreted as points of possible recombination between the input genomes.

Graph topology is also constrained by our requirement that the constructed graph be, for interoperability reasons, representable in VCF format; that is, it must not contain cycles, and it has to contain a separate connected component for each chromosome. Some third-party inference methods support fully general VCFs with overlapping variant alleles; other frameworks, for example gramtools ( Maciuca *et al.*, 2016), require that the encoded variants be non-overlapping. To achieve full interoperability with different downstream inference methods, NovoGraph therefore implements two separate algorithms for VCF generation: NovoGraph-Simple, which outputs a VCF which may contain overlapping variant alleles, and NovoGraph-Universal, which outputs a VCF with non-overlapping variant alleles.

The first of these algorithms, NovoGraph-Simple, implements a one-to-one conversion of each aligned contig from the multiple sequence alignment into VCF format. Given a pairwise alignment between the canonical reference and the contig, let *ALIGNED_REF* and *ALIGNED_CONTIG* refer to the two aligned sequences (i.e., including gap characters). We initialize two variables *RUNNING_REF* and *RUNNING_CONTIG* as empty strings. We then iterate through the pairwise alignment in a column-by-column fashion from left to right; for each column *i* of the pairwise alignment, we perform the following steps:

1. If *ALIGNED_REF[i] ≠ ALIGNED_CONTIG[i]*, continue to step 2. Otherwise, carry out the following: a) remove all gap characters from *RUNNING_REF* and *RUNNING_CONTIG*; b) if *RUNNING_REF* and *RUNNING_CONTIG* are then not identical, output a VCF variant with reference sequence *RUNNING_REF* and variant sequence *RUNNING_CONTIG*; c) reset *RUNNING_REF* and *RUNNING_CONTIG* to the empty string.
2. Append the character *ALIGNED_REF[i]* to *RUNNING_REF*, and append *ALIGNED_CONTIG[i]* to *RUNNING_CONTIG*. Then, if the end of the pairwise alignment has not been reached, return to step 1 with *i = i + 1.*

If necessary, the pairwise alignments are pruned so that the first and the last column are non-gap and reference-identical; no further regularization (other than that carried out implicitly by the employed MSA algorithm) of alignment gap structure is carried out. After having applied the conversion algorithm to all aligned contigs, identical variants from different contigs are merged and sorted by position to obtain a valid VCF. This algorithm implements a merging model that allows for transitions between pairs ( *a*, *b*) of MSA sequences immediately prior to positions at which both *a* and *b* align to the reference in the joint MSA with either a match or a mismatch. The generated VCF may contain overlapping variants.

The second graph generation algorithm, NovoGraph-Universal, is more complex and ensures that the created VCF does not contain overlapping variant alleles; that is, it ensures universal compatibility with third-party inference methods.

NovoGraph-Universal allows for the merging at input sequences (A) between pairs of input contigs wherever input contigs start or end along the canonical reference, and (B) at positions at which all contig sequences agree with the canonical reference. The graph collapses into a uniformly homozygous state at positions whereby condition (B) applies. The resulting graph structure (composed of reference-identical, collapsed stretches interspersed with sets of alternative haplotypes) lends itself directly to representation in VCF format. Also note that criterion B (sequence identity across all input sequences) is stronger than the merging condition (sequence identity across pairs of input sequences) of a related algorithm ( Dilthey *et al.*, 2015).

**Figure 4.**
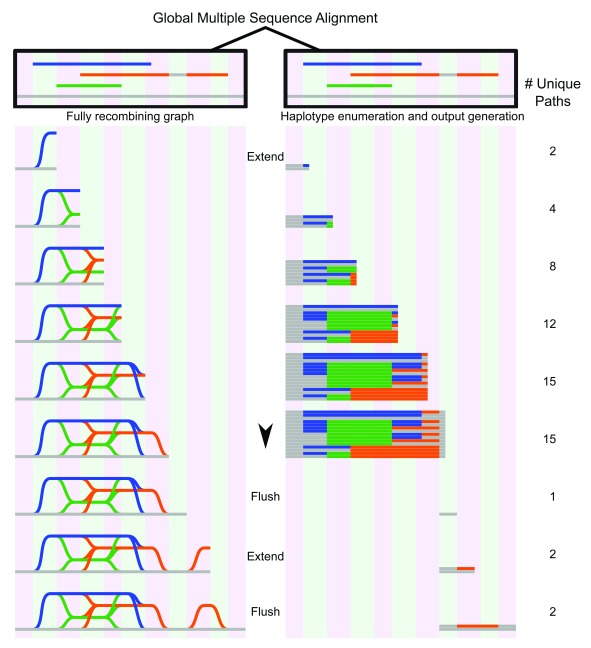
Graph genome generation with NovoGraph-Universal. Graph representation (left) and unique variants (right) produced by graph genome alignment. From the global multiple sequence alignment, all unique paths through the graph genome are enumerated and written to output. In this example, the reference genome (gray) serves as a scaffold to which all contigs (blue, green, and orange) are aligned. In the first “extension” phase, all unique paths through the graph are identified until deviation from reference genome terminates. At this point, all variant paths are output, or “flushed” to the genome graph output; in this implementation, the variants are written to a VCF file. In the second extension phase, the orange contig deviates again from the reference genome, producing another variant, which, following coalescence back to the reference genome, is “flushed” to the output file.

An overview of NovoGraph-Universal is given in Figure 4. At a high level, NovoGraph-Universal constructs a graph by processing the input MSA for each reference contig in a column-by-column fashion from left to right in the order of genomic position, accounting for the entry and exit of input contigs as well as for potential transitions between them. This can be viewed as a breadth-first exploration of the MSA. As the algorithm moves along the MSA, it keeps track of the set of haplotypes compatible with the input contigs and their potential recombinants. In the graph, each haplotype is generally represented as its own branch; however, these are collapsed at positions at which all haplotypes agree with the canonical reference. The sequences corresponding to this “collapsed homozygous” state are reference-identical and therefore not explicitly represented in VCF.

In the following, we provide a more detailed description of the algorithm:

NovoGraph-Universal is executed for each GRCh38 reference contig independently. The set of input sequences for each reference contig is represented by the multiple sequence alignment constructed during Step 2. Each non-reference contig in the MSA has a first and a last column in which both the input contig and reference bases are non-gap. We refer to these columns as the entry and exit positions of the contig, and all bases outside the entry and exit columns are ignored during the following steps.

We keep a set of current haplotypes, denoted as *R*. Each element *h* of *R* (a “current haplotype”) consists of two elements: (i) the “current sequence” of *h* (which is updated as we move along the MSA) and (ii) the contig ID of the contig that the current haplotype is copying from (the “source haplotype”)—this can be either the reference or one of the input contigs. We initialize *R* such that *R* has one element that has a zero-length sequence and that is set to copy its sequence from the reference.

When we process a column of the MSA, for each element *h* ∈ *R*, we append the corresponding MSA character of the source haplotype to the current sequence of *h*. This step is called “extension” (see Figure 4).

After having carried out the extension step, the current sequences of the elements of *R* up to the second-last character are sent to the VCF generator if and only if (A) all appended characters are non-gap reference identical and (B) the length of the current sequences of the elements of *R* is greater than or equal to 2 non-gap bases. This step is called “flushing” (see Figure 4). The VCF generator writes a variant-encoding line to the output VCF file if the received sequences contain at least one non-reference sequence; otherwise, the output is empty. After processing by the VCF generator, the processed strings are removed from their corresponding source elements—that is, after flushing, the current sequences of all elements of R have a length of 1 (the last added base, which was not sent to the VCF generator). Note that *R* is a set so that, by definition, duplicate elements are collapsed. This process is carried out up to the rightmost column of the MSA, at which point the graph construction and VCF generation process is complete.

Two special cases corresponding to the entry and exit of non-reference contigs conclude the definition of the graph construction algorithm. First, if an MSA position being processed corresponds to the entry position of a contig, we duplicate all elements of *R* prior to the extension step, set the source haplotype of the duplicate elements to the ID of the starting contig, and add the modified duplicates to *R*. Second, if an MSA position being processed corresponds to the exit position of a contig, we execute the following algorithm after the extension step:

**1.**  Compile a list *E* of all elements of *R* which use the existing contig as their source haplotype (i.e. the elements *R* of affected by the contig exit).
**2.**  Compile a list *C* of non-reference contig IDs that a) are the source haplotype of any current element in *R* and b) don’t exit at the current MSA position (i.e. *C* is a list of non-exhausted current contig IDs).
**3.**  For each element *(e, c)* ∈ { *E* x *C*}, we add a new element to *R* with a) its current sequence set to the current sequence of *e* and b) its source haplotype set to *c*. After having processed all elements of the set { *E* x *C*} we set the source haplotypes of all *e E* to the reference.

Clearly the size of *R* increases as non-reference contigs enter and exit and, conversely, the size of *R* can only decrease during the flushing step. To limit computational demands, we impose an upper limit *U1* on the size of *R*. If *|R|* ≥ *U1*, we prohibit the entry of new contigs, and when exiting a contig, we only allow the transition to the reference as source haplotype. If the entry of a contig is prohibited, the contig is lost permanently (including the variants it contains).

Furthermore, due to the requirement of reference identity, gaps in the input MSA along the contig sequence dimension (i.e. corresponding to columns in the MSA in which the input contig sequence is a gap and the reference is not) prevent flushing. We therefore also place an upper limit *U2* on the maximum number of contiguous contig gaps in the input alignments. If a contiguous gap along the input contig dimension in an input contig alignment exceeds *U2* in size, we break the alignment, i.e. we split the alignment in two. *U1* limits the complexity of the graph in terms of the number of per-site variant haplotypes, *U2* limits the maximum size of deletions represented in the graph. In the current implementation, we use *U1 = U2 =* 5000 bp, but both parameters can be easily modified by the user.

### Implementation and computational requirements

Steps 1 and 2 are implemented in Perl 5. Step 3 is implemented in C++, with a wrapper Perl script. Our pipeline utilizes bwa (version 0.7.15 and above), SAMtools (version 1.4 and above), and MAFFT (version 7). The minimum computational requirement for NovoGraph is a workstation computer with at least 32 Gb of RAM; we recommend, however, that the MSA generation steps be executed within a multi-node cluster environment. NovoGraph natively supports SGE-compatible grid environments, although this could be easily adapted to other platforms.

### Human input assemblies

**Figure 5.**
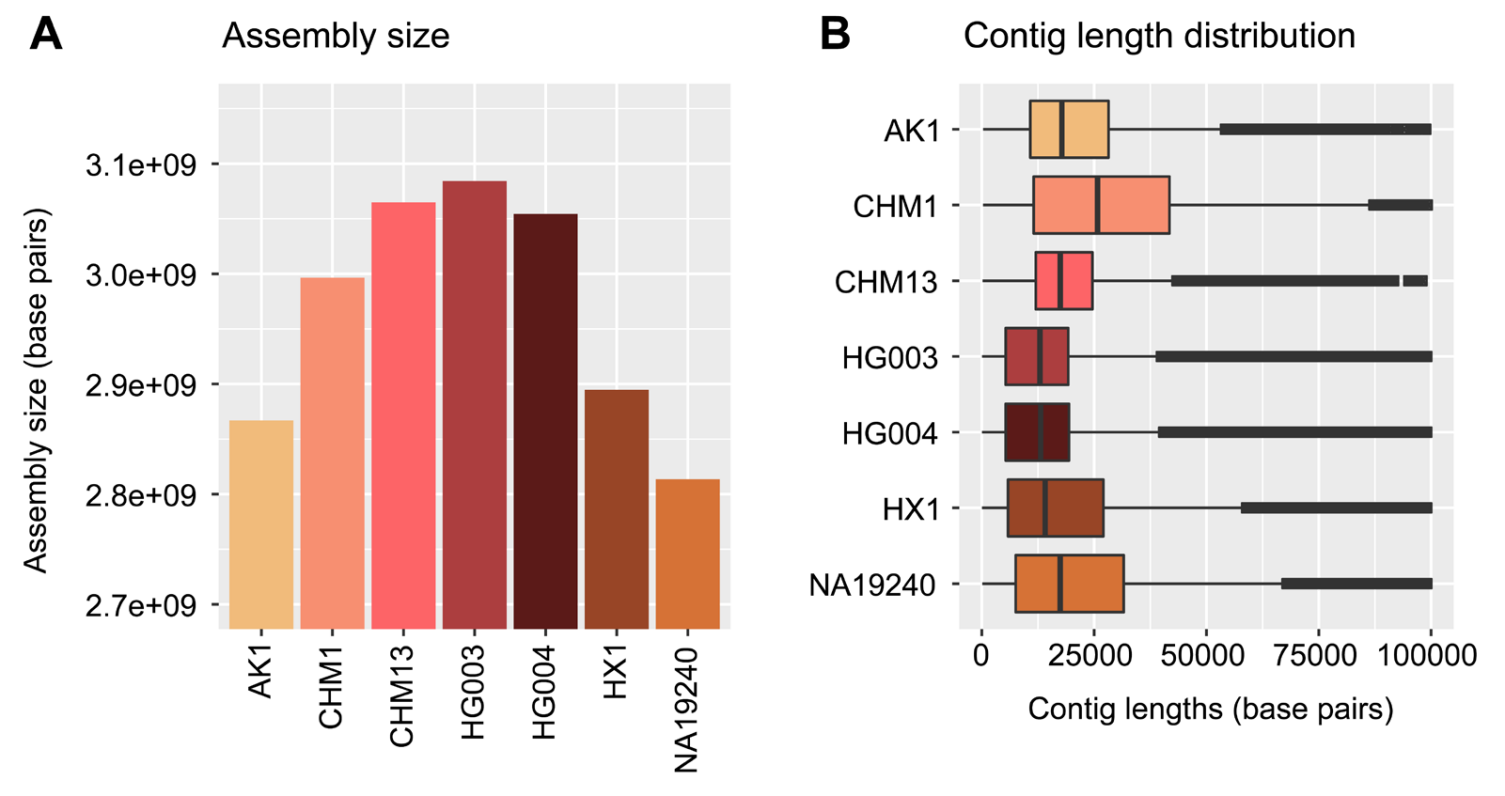
Assembly sizes and contig lengths. Assembly sizes ( **A**) and contig length distributions ( **B**) shown in units of base pairs for each input human assembly (see Table 1) used to demonstrate NovoGraph.

**Figure 6.**
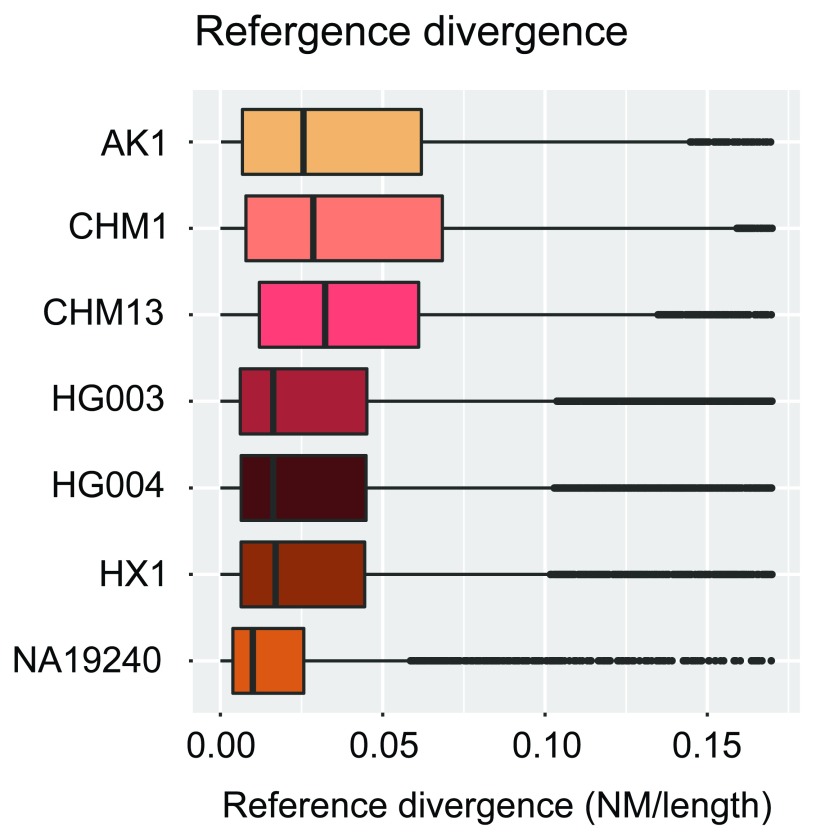
Reference divergence per input assembly. Reference divergence (edit distance divided by contig length; see text) for each contig within each individual assembly. No effort was made to classify variants within each assembly as genuine variation or errors.

We used contigs from seven recent *de novo* assemblies of human genomes ( Table 1), the data of which are publicly available. The total size and contig lengths of each input assembly are shown in Figure 5. In order to quantify the sequencing and alignment quality of each input assembly, we relied upon the edit distance (Levenshtein distance) encoded via the BAM NM tag, i.e. the number of nucleotide changes within each contig necessary to equal the reference. The results of dividing this value by the length of each aligned contig (NM/Length) are shown in Figure 6. We note that we have made no effort to classify variants within each assembly as genuine variation or errors.

**Table 1.** Input assemblies for the whole-genome human graph.

Sample ID	Ethnicity	Citation	Download URL
AK1	Korean	( Seo *et al.*, 2016)	https://www.ncbi.nlm.nih.gov/Traces/wgs?val=LPVO02#contigs
CHM1	European	( Chaisson *et al.*, 2015; Steinberg *et al.*, 2014)	https://www.ncbi.nlm.nih.gov/assembly/GCA_001297185.1/
CHM13	European	( Schneider *et al.*, 2017)	https://www.ncbi.nlm.nih.gov/assembly/GCA_001015385.3
HG003	Ashkenazi	( Zook *et al.*, 2016)	ftp://ftp-trace.ncbi.nlm.nih.gov/giab/ftp/data/AshkenazimTrio/ analysis/MtSinai_PacBio_Assembly_falcon_03282016/hg003_ p_and_a_ctg.fa
HG004	Ashkenazi	( Zook *et al.*, 2016)	ftp://ftp-trace.ncbi.nlm.nih.gov/giab/ftp/data/AshkenazimTrio/ analysis/MtSinai_PacBio_Assembly_falcon_03282016/hg004_ p_and_a_ctg.fa
HX1	Han Chinese	( Shi *et al.*, 2016)	http://hx1.wglab.org/data/hx1f4.3rdfixedv2.fa.gz
NA19240	Yoruba	( Steinberg *et al.*, 2016)	https://www.ncbi.nlm.nih.gov/assembly/GCA_001524155.1/

### vg mapping experiment

We used the variation graph toolkit vg ( Garrison *et al*., 2018) to assess the effect of mapping against the constructed human genome graph (based on the NovoGraph-Universal algorithm). Short-read sequencing data of sample NA12878 were obtained from the Platinum Genomes project (2 x 100bp paired-end sequencing reads; European Nucleotide Archive accession ERR194147) and randomly subsampled to 2% of read pairs. We mapped the subsampled reads to the genome graph constructed by us and against a genome graph constructed from the GRCh38 primary reference and assessed the resulting alignment metrics (alignment score, alignment identity, number of mapped reads).

## Results

We have presented NovoGraph, a pipeline for the construction of genome graphs from *de novo* assemblies and applied the pipeline to construct a genome graph from seven high-quality, ethnically diverse human assemblies ( Biederstedt, 2018). 19 out of 63185 input contigs failed to generate initial alignments with bwa-mem and were therefore ignored for all further steps. The majority of these contigs are very short (<200 bases). The graph constructed by NovoGraph-Universal has a size of 17 Gb when stored in uncompressed VCF format and contains 23,478,835 bubbles (i.e. sites with multiple alternative alleles) representing 30,582,795 variant alleles. The graph constructed by NovoGraph-Simple has an uncompressed size of 1.2 GB in VCF format and contains 33,309,666 bubbles representing 34,519,145 variant alleles. Both graphs and intermediate files are available for download and can be used for genome inference with a variety of tools.

**Figure 7.**
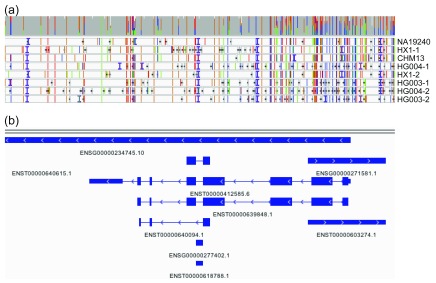
IGV visualization of *HLA-B*. The *HLA-B* region for the genome graph produced by our approach as visualized in the Integrated Genomics Viewer. ( **a**) The coverage (gray bar) of the eight included assemblies (NA19240, HX1, etc.) and the alignment of each to the graph genome. Colored vertical lines indicate sequence variants (green = A, blue = C, orange = G, red = T), horizontal black lines indicate deletions, and vertical purple “I” characters show insertions. ( **b**) Genomic annotations. High rates of polymorphism are observed around peptide-binding-site encoding exons 2 and 3.

We manually assessed a small set of hyperpolymorphic regions in the human genome. Figure 7 shows an IGV-based visualization ( Robinson *et al.*, 2011; Thorvaldsdóttir *et al.*, 2013) of the multiple sequence alignment of the input sequences in the *HLA-B* region of the MHC. *HLA-B* is the most polymorphic gene of the human genome and sequence polymorphisms are known to cluster around the peptide-binding-site encoding exons 2 and 3 ( Marsh *et al.*, n.d.); consistent with this, high rates of polymorphism are observed in our multiple sequence alignment around these loci.

To measure the extent to which mapping against the constructed graph influences alignment metrics, we used the variation graph toolkit vg to map a randomly selected subset of NA12878 reads (see Methods) against a) the genome graph constructed by us (based on the NovoGraph-Universal algorithm) and b) a simple non-branching reference graph constructed from the primary GRCh38 reference alone. Alleles longer than 10 kb in size were removed to ensure successful loading of the graphs into vg. Results of the mapping experiment are shown in Table 2; while mean alignment identity is increased by approximately 0.2%, the number of mapped reads decreases by 0.04%. This somewhat counterintuitive result is probably explained by greater alignment ambiguity for a subset of reads, caused by the presence of non-unique branches in the graph; reads with multiple optimal mapping locations will be assigned a mapping quality score of 0 and count as unmapped.

**Table 2.** Read alignment quality metrics for the NA12878 mapping experiment. A total of 2% of NA12878 Illumina Platinum reads were mapped against the NovoGraph-constructed genome graph (“Genome graph”) and against a GRCh38-equivalent genome graph (“Reference graph”; no ALT contigs used). As expected, mapping against the genome graph increases mean alignment scores and alignment identities, albeit at a small reduction in the number of mapped reads.

	Genome graph	Reference graph
Mean scores	108.859	108.100
Mean identity value	0.9913	0.9891
Total mapped reads	31125004	31138410

## Conclusion and next steps

NovoGraph enables the construction of a graph genome from multiple *de novo* assemblies. The pipeline is available under an open source license and will scale to at least a few dozen input assemblies without major modifications. It would also be straightforward to adapt NovoGraph to non-human species, given the appropriate reference and input assemblies.

It is instructive to contrast the MSA-based NovoGraph approach with possible alternative approaches in which one creates a separate VCF for each assembly and then builds a graph by combining the individual VCFs. First, carrying out the multiple sequence alignment prior to the VCF generation step enables the sharing of information across multiple samples during the alignment process, potentially improving overall alignment quality and providing more consistent variant definitions across samples. Secondly, the constructed multiple sequence alignment of all input assemblies can be repurposed for other applications, for example as an input to other graph construction algorithms like the Population Reference Graph ( Dilthey *et al.*, 2015). Finally, as the number of input genomes increases in size, it will become increasingly necessary to establish the mutual homology relationships between variant alleles from different samples and to represent these in the form of nested graphs; the MSA contains the information necessary for this. As an example, consider the case of two large insertion variants that differ from each other by a single base: in the field of graph genomes, these are most naturally represented as one large insertion with an additional SNP nested into it (instead of two near-identical branches). These points notwithstanding, multiple sequence alignments come at a computational cost, and might prove to be computationally prohibitive if the number of input genomes increases by more than one order of magnitude.

One limitation of NovoGraph (and existing approaches for downstream graph-based genome inference) is that no attempt is made to explicitly model or annotate events that break co-linearity between the input sequences, such as rearrangements, inversions, or translocations. If present, the corresponding sequences will be represented in the MSA and feature as bubbles in the generated graph, so that downstream inference on the presence or absence of these features is possible in principle. Meaningful integration of complex variant types into models for graph-based genome inference, however, will require further work in methods development. This is an important direction for future research.

There are two additional directions for future work. First, in the spirit of a hackathon, we have focused our efforts on the software development process. A comprehensive empirical evaluation of the constructed human genome graph is still outstanding. This could be achieved by loading the graph into multiple graph-based inference frameworks and by measuring genome inference accuracy. Secondly, it would be important to better understand the impact on the graph construction process of various parameter settings and trade-offs. For example, in the interest of simplicity, we implemented a simple gap scoring scheme that is neither affine nor convex; we relied on the default settings of MAFFT for the generation of the multiple sequence alignments; and we implemented a naive algorithm to split the reference genome into windows for MSA generation. Exploring alternative choices in each of these cases would be straightforward and could lead to valuable insights. A convex gap scoring scheme would probably improve the alignment of large and complex structural variants ( Sedlazeck *et al.*, 2018) and therefore be the most important point to address.

These limitations notwithstanding, we believe that NovoGraph represents a useful addition to the field of graph genomes. A strength of NovoGraph is its ability to generate genome graphs for all major genome graph approaches directly from *de novo* assembly data. The graphs constructed with NovoGraph are available for download and could, for example, inform comparisons of different genome graph construction methods and the improved calling of structural variation.

## Data availability

Input assemblies are publicly available and carry the NCBI assembly accession numbers GCA_001750385.2 (AK1), http://identifiers.org/ncbigi/GI:1078263188; GCA_001297185.1 (CHM1), http://identifiers.org/ncbigi/GI:929855629; GCA_001015385.3 (CHM13), http://identifiers.org/ncbigi/GI:953917559; GCA_001549605.1 (HG003), http://identifiers.org/ncbigi/GI:985741195; GCA_001549595.1 (HG004), http://identifiers.org/GI:985734877; GCA_001524155.1 (NA19240), http://identifiers.org/ncbigi/GI:1057722128; GCA_001708065.2 (HX1), http://identifiers.org/ncbigi/GI:1087879108. Full assembly data access details are given in Table 1.

All NovoGraph output data are available on OSF: https://doi.org/10.17605/OSF.IO/3VS42 ( Biederstedt, 2018). The genome graphs of seven ethnically diverse human genomes in VCF format can be downloaded from https://osf.io/t5czk/?view_only=fedd8437d96c4d688f6c40150903d857 (constructed with NovoGraph-Universal) and https://osf.io/pgq52/?view_only=fedd8437d96c4d688f6c40150903d857 (constructed with NovoGraph-Simple). The global multiple sequence of all input sequences in CRAM format can be downloaded from https://osf.io/jhbwx/?view_only=fedd8437d96c4d688f6c40150903d857. OSF data are available under the terms of the Creative Commons Zero "No rights reserved" data waiver (CC0 1.0 Public domain dedication).

## Software availability

Source code for the pipeline is available from: https://github.com/NCBI-Hackathons/NovoGraph.

Archived source code at time of publication: https://doi.org/10.5281/zenodo.1342485 ( Biederstedt *et al*., 2018).

License: MIT license.
