# Supplementary material for: NovoGraph: Human genome graph construction from multiple long-read de novo assemblies
Source: F1000Res. 2018 Dec 10;7:1391. Originally published 2018 Sep 3. [Version 2] doi: 10.12688/f1000research.15895.2 (PMC6305223; doi:10.12688/f1000research.15895.2)
Supplement: Peer review history

# Peer review: Referee response for version 2

**DOI:** 10.5256/f1000research.18946.r41684

**Published:** 2018-12-19

**Authors:** Korbinian Schneeberger, Manish Goel

**Affiliations:** 1 Department of Plant Developmental Biology, Max Planck Institute for Plant Breeding Research, Cologne, Germany

Thank you for considering our comments. I support the peer-reviewed publication of this manuscript.

We have read this submission. We believe that we have an appropriate level of expertise to confirm that it is of an acceptable scientific standard.

# Peer review: Referee response for version 1

**DOI:** 10.5256/f1000research.17353.r38468

**Published:** 2018-10-11

**Authors:** Korbinian Schneeberger, Manish Goel

**Affiliations:** 1 Department of Plant Developmental Biology, Max Planck Institute for Plant Breeding Research, Cologne, Germany

The authors present NovoGraph a method to build up a genome graph from whole-genome alignments between reference genome and de novo assemblies. Instead of building up a genome graph similar to an assembly graph, the genomes are assembled separately and then integrated into a graph based on reference-based whole-genome alignments. Practical application of NovoGraph is shown with a genome graph build up from seven human genome assemblies. The tool targets a practical problem filling a gap in a workflow with otherwise existing software. I would consider this idea as broadly useful and applicable in special cases. A main critic for this work would be its lack of scalability and flexibility (addition/removal of genomes) which might limit its applicability. The manuscript is well and clearly written.

 Major (which the authors should consider)

1.  In the title, the authors state that the method is applicable for “long-read de novo assemblies”. Are there any specific reasons that the authors decided to restrict the scope of their method?
2. The method is geared to human genome comparison. This should be made clear in the title and abstract to avoid confusion as it might not be so straightforward to adapt it to other species if type and degree of sequence variation is different (see point 3 and 4). Otherwise, the authors could demonstrate how to adjust the tool for other genomes with higher degree of structural variation as well.
3. If a “consistent global alignment” of a contig (step 1) can only be on one reference contig and can only consider one alignment direction, how are inversion breakpoints and cross-chromosome translocation breakpoints identified?
4. How are large insertions (i.e. sequences not present in the ref seq) represented in step 2? How are the per-window MSA combined if different genomes have different orders of these 10kb windows (e.g. in translocations, inversions…)?
5. Were there contigs that were too divergent to be aligned in the test cases? What if those exist?
6. The authors mentioned that the graph construction is constrained by their requirement to generate VCF files, however, they don’t mention what could be the potential effects of this restriction.
7. The authors have cited work which has not yet been published after peer review. Is this common for F1000research, if not, please mention that these citations are non-peer-reviewed preprints.
8. In order to limit computation load, the method uses hard cut-offs for the number of haplotypes that can be analysed simultaneously. Consequently, new contigs are not added. However, the authors do not describe what happens to those contigs. Are they removed permanently? If yes, then would that lead to potential loss of alternate haplotypes that could be identified from available data?

Minor

1. We agree with the other reviewer that the authors might want to consider the well-established definitions of “recombination” and “homologous recombination” and perhaps try to find different wording for the branching points in the graph. It is not clear what “homologous-identical recombination” (abstract) or “homologous identical positions” (methods).
2. Similarly, it is conventional to write Directed Acyclic Graph instead of acyclic directed graph, and the authors might want to change that.
3.  “uneven” => “odd”?
4. Figure 2 could be extended with cases where  “entry” and “exit” points are within alignments as well.

We have read this submission. We believe that we have an appropriate level of expertise to confirm that it is of an acceptable scientific standard, however we have significant reservations, as outlined above.

# Peer review: Referee response for version 1

**DOI:** 10.5256/f1000research.17353.r38774

**Published:** 2018-10-03

**Authors:** Bjarni V. Halldorsson

**Affiliations:** 1 deCODE Genetics/Amgen, Inc., Reykjavik, Iceland

The manuscript describes a method to create a graph genome from a set of input sequences, such as whole genome assembly. This is a topic of current interest to the community and I am not aware of another implementation for this problem. The paper implementation is practical and likely to be useful, but it is also clear that that there is considerable room for improvement and work in this field.

 The provide an algorithm and software. The algorithm has three steps: 1) Whole genome alignment 2) Local multiple sequence alignment 3) Graph construction/VCF construction from graph.

 There is a large body of literature on whole genome alignment which might be cited and explained to the reader what the difference is between the current method and previously described ones.

 Multiple sequence alignment is also a well studied problem, this literature might be cited and it is not clear why MAFFT was chosen over other implementations.

 I would suggest the authors find another word to replace “recombination” in their text, recombination has a well defined meaning in genetics/meiosis which to me seems to be different from the meaning the authors assign to the word. The authors say the branching points in the graph are recombination points between the input genomes, but they might as well be due to SVs.

 Why do the authors choose to claim that the human reference genome was completed in 2003? There have been a number of updates since then and the reference genome is still filled with gaps. The authors are using GRCh38 which was released in December 2013.

 Genomic sequencing can be considered to be a key tool in research since shortly after invented, certainly since long before 2003.

 On page 4, step 2 states that a global MSA is computed, but from figure 2 it is clear that this is in fact a series of local MSAs.  I would suggest using another term than global alignment or at least refer to it as inexact/approximate global MSA.

 From figure 1, step 2.1 it might be inferred that identifying windows was a more involved process than partitioning the genome into 10kb windows.

 I am not sure how NovoGraph-Simple is implemented, the details are not described in the manuscript. For NovoGraph-Simple to be useful, it would be very useful if the “The positions where an input sequence deviates from the reference is represented as variant alleles in a valid VCF” was better defined.  For inserted sequence, particularly microsatellites/STRs, there are multiple valid VCF records that can be created. Conventions such as left aligning would be useful for the user.

 If I understand correctly, NovoGraph-Universal is a BFS (breadth first search) of the graph. The authors might make a note of this.

 It is clear that NovoGraph-Universal is not a practical implementation for people studying a large number of genomes. Whenever a single sequence diverges from the reference all sequences are expanded. Once enough individuals have been sequenced this will lead to VCF files where each individual's’ chromosomes are the alternate allele.

 I have only evaluated the manuscript and not the software or the VCF files.

I have read this submission. I believe that I have an appropriate level of expertise to confirm that it is of an acceptable scientific standard.
